# Sclerostin is a promising therapeutic target for oral inflammation and regenerative dentistry

**DOI:** 10.1186/s12967-022-03417-4

**Published:** 2022-05-13

**Authors:** Chufang Liao, Shanshan Liang, Yining Wang, Ting Zhong, Xiangning Liu

**Affiliations:** 1 School of Stomatology, Jinan University, Guangzhou, China; 2 Clinical Research Platform for Interdiscipline of Stomatology, Jinan University, Guangzhou, China; 3 Department of Stomatology Medical Center, The First Affiliated Hospital of Jinan University, Guangzhou, China; 4 The State Key Laboratory Breeding Base of Basic Science of Stomatology (Hubei-MOST) & Key Laboratory of Oral Biomedicine Ministry of Education, School & Hospital of Stomatology, Wuhan University, Wuhan, China; 5 Department of Prosthodontics, Hospital of Stomatology, Wuhan University, Wuhan, China

**Keywords:** Sclerostin, *SOST*, Periodontitis, Dental pulp stem cells, Dentinogenesis, Dental implant, Alveolar bone

## Abstract

Sclerostin is the protein product of the *SOST* gene and is known for its inhibitory effects on bone formation. The monoclonal antibody against sclerostin has been approved as a novel treatment method for osteoporosis. Oral health is one of the essential aspects of general human health. Hereditary bone dysplasia syndrome caused by sclerostin deficiency is often accompanied by some dental malformations, inspiring the therapeutic exploration of sclerostin in the oral and dental fields. Recent studies have found that sclerostin is expressed in several functional cell types in oral tissues, and the expression level of sclerostin is altered in pathological conditions. Sclerostin not only exerts similar negative outcomes on the formation of alveolar bone and bone-like tissues, including dentin and cementum, but also participates in the development of oral inflammatory diseases such as periodontitis, pulpitis, and peri-implantitis. This review aims to highlight related research progress of sclerostin in oral cavity, propose necessary further research in this field, and discuss its potential as a therapeutic target for dental indications and regenerative dentistry.

## Background

The *SOST* gene was initially identified in the late 1990s during the studies on sclerosteosis and Van Buchem disease, which are two rare hereditary diseases characterized by high bone mass [1, 2]. Sclerostin, the protein product of the *SOST* gene, is a well-known osteogenesis inhibitor that plays a vital role in bone remodeling [3–6]. A humanized monoclonal sclerostin-neutralizing antibody (Scl-Ab) called romosozumab was approved in 2019 by the Food and Drug Administration (FDA) for treating osteoporosis in patients with a high risk of fracture, following years of clinical trials evaluating the pharmacology, efficacy, and safety [7]. In recent years, sclerostin has received much interest from the researchers in the oral field. Furthermore, sclerostin has been found to participate in dentinogenesis, cementogenesis, alveolar bone remodeling, pupal/periodontal inflammation and implant osseointegration. This review aims to highlight the role of sclerostin in the formation and maintenance of tooth and periodontal supporting tissues, suggest more specific scientific evidence in this field, and prospect potential therapeutic applications for dentistry.

A PubMed and Google Scholar online database search for relevant studies was performed in October 2021 and updated in April 2022. Search terms included “sclerostin”, “*SOST*”, and terms of dental tissues, periodontal structures, oral cell types and dental diseases. Articles were screened by title and abstract. Evidence was acquired from in vivo and in vitro experimental studies and clinical trials.

## Sclerostin: a molecule that regulates bone metabolism

The *SOST* gene is located in the human chromosomal region 17q12-q21, with only two exons and one intron [8, 9]. Six mutants of the *SOST* gene have been reported: three premature termination codons, two splice site mutations, and one missense mutation [10, 11]. Sclerosteosis is caused by the mutation of *SOST* gene [9], whereas Van Buchem disease results from the deletion of a 52-kb fragment containing an enhancer element downstream of the *SOST* transcription start site [12, 13]. These two bone dysplasia diseases are similarly characterized by generalized hyperostosis or sclerosis of the skull and even all long bones due to sclerostin protein deficiency.

**Fig. 1:**
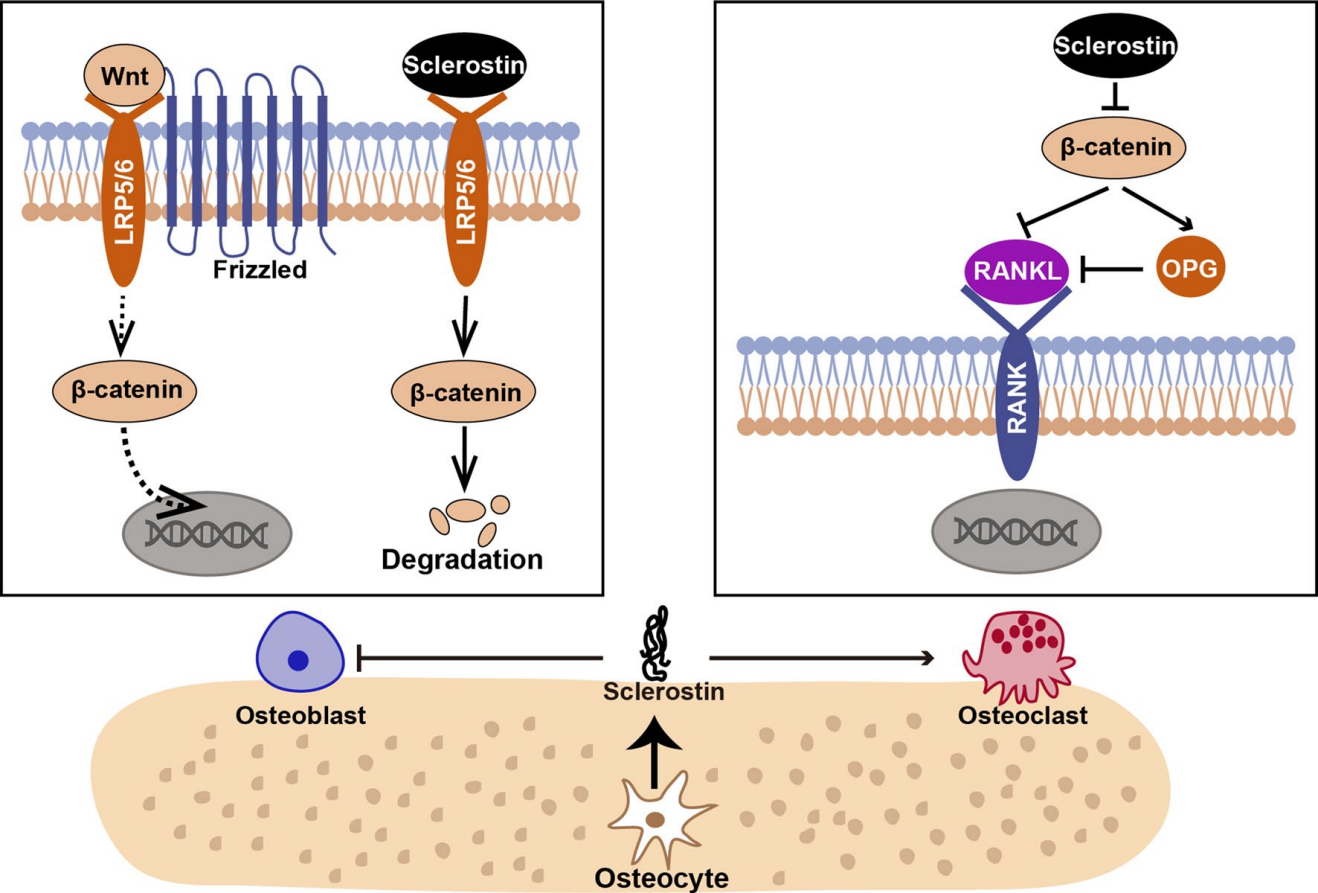
Role and mechanisms of sclerostin secreted by osteocytes in bone metabolism. Sclerostin inhibits Wnt/β-catenin pathway by competitively binding to LRP5/6, promoting the ubiquitinated degradation of β-catenin and blocking its nuclear import to inhibit the osteogenic function of osteoblasts. Besides, sclerostin increases the production of RANKL while decreases the production of OPG in osteoblasts and pre-osteoblasts to active the osteoclasts. *LRP5/6* low-density lipoprotein receptor protein 5/6; *RANKL* receptor activator of nuclear factor-κB ligand; *OPG* osteoprotegerin; *RANK* receptor activator of nuclear factor-κB

Sclerostin is a secretory glycoprotein containing 213 amino acids with a relative molecular weight of 40 kDa. The amino acid sequence of sclerostin in humans has 88% and 89% homology with mice and rats, respectively. Sclerostin expression can be modulated by multiple factors such as mechanical stress, oxygen, hormones, and transcription factors [14–17]. Sclerostin potently inhibits bone formation by regulating the proliferation, differentiation, mineralization, and apoptosis of pre-osteoblastic cells and osteoblasts [18–20]. Sclerostin deficiency causes high bone mass in humans and experimental animals [3, 9, 12]. In contrast, sclerostin overexpression in mice causes a remarkable reduction in bone mass and volume [18, 21]. Sclerostin contains a cysteine knot, and the knot motif shares 20–24% similarity with differential screening-selected gene aberration in neuroblastoma (DAN) protein family also containing a cystine knot [8, 22]. The DAN protein has been shown to inhibit osteogenesis by antagonizing bone morphogenetic proteins (BMPs). Thus, early studies presumed that sclerostin also served as a BMPs antagonist to inhibit bone formation [8, 18]. However, subsequent studies have confirmed that the negative effects of sclerostin on osteogenesis were mainly achieved by blocking the Wnt/β-catenin signaling pathway rather than the BMPs pathway. The Wnt proteins are crucial for skeletal formation and development. Sclerostin competitively binds to the low-density lipoprotein receptor protein 5/6, decreasing its combination with Wnt proteins and Frizzled receptor, blocking the activation of Wnt/β-catenin pathway and consequently inhibiting bone formation (Fig. 1) [19, 23, 24]. Moreover, several studies have proposed that sclerostin also plays an essential role in bone resorption. Receptor activator of nuclear factor-κB ligand (RANKL) is the key factor for osteoclast differentiation and activation. Osteoprotegerin (OPG) is the decoy receptor of RANKL that downregulates bone resorption. Sclerostin can promote osteoclast production and activate osteoclast function by regulating the RANKL/OPG axis (Fig. 1) [21, 25].

Advanced knowledge about sclerostin's negative role in bone mass was adapted from the laboratory experimentation to the clinic. Romosozumab, a humanized monoclonal Scl-Ab, has been approved as an alternative therapy for postmenopausal women with a high risk of osteoporotic fractures, and is expected to become a novel method for accelerating fracture healing and treating sclerosteosis and myeloma bone disease [24, 26–29].

## Distribution of sclerostin in oral tissues and cells

It has been recognized that sclerostin has been exclusively expressed in the bone tissue for a long time. Sclerostin is mainly secreted by osteocytes and, to a lesser degree, by chondrocytes and osteoclasts [18, 20, 22]. However, patients with sclerosteosis or Van Buchem disease also display some dental malformations, such as malocclusion, partial anodontia, delayed tooth eruption, and abnormal tooth shape and position [30, 31]. These manifestations due to sclerostin deficiency inspired the investigation of sclerostin in the oral field.

**Fig. 2:**
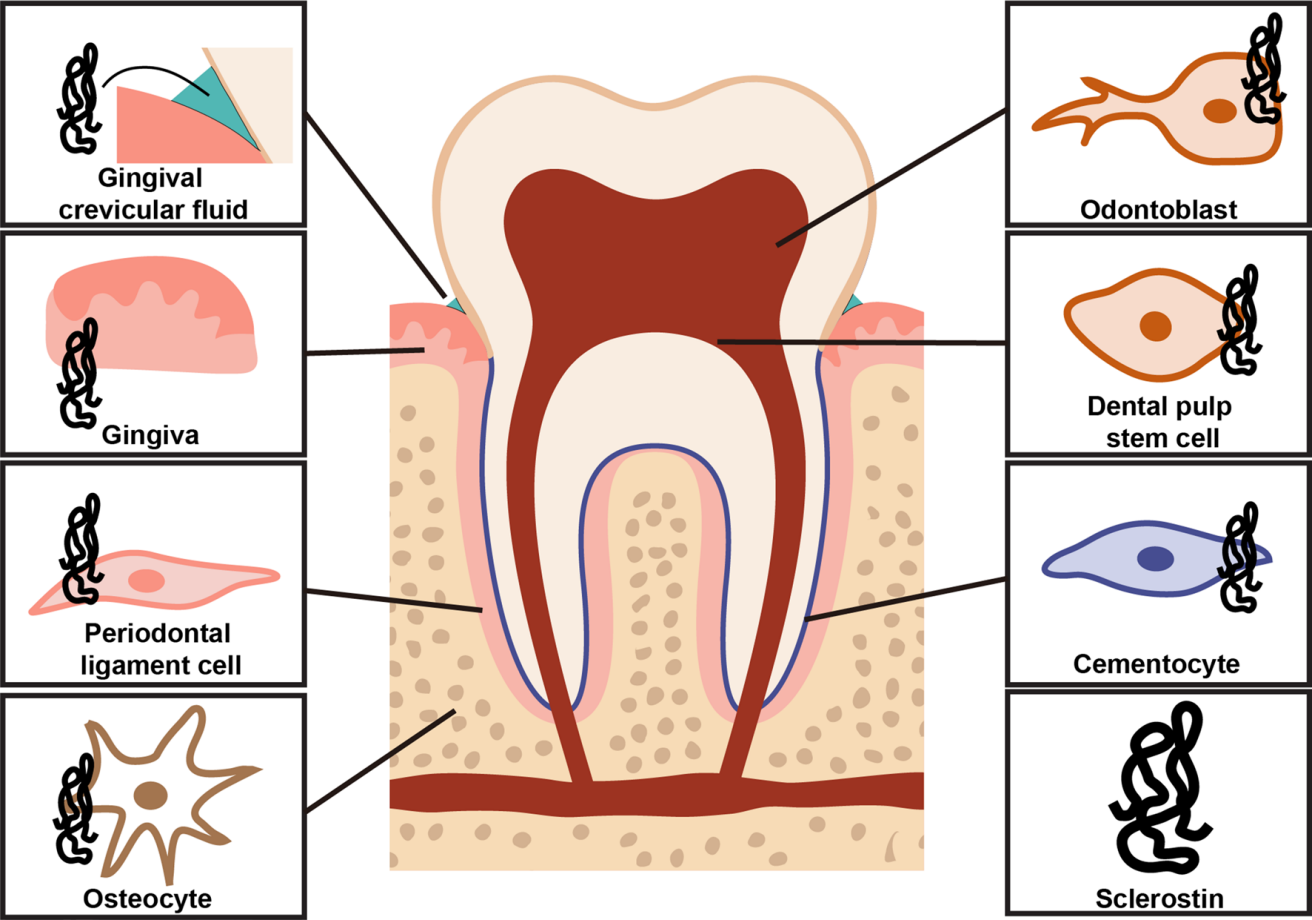
The distribution of sclerostin in oral tissues and cells. Sclerostin is distributed in gingiva and gingival crevicular fluid, periodontal ligament cells and osteocytes in periodontal supporting tissues, odontoblasts and dental pulp stem cells in dental pulp, and cementocytes in cellular cementum

There are many cell types in dental and periodontal tissues, with different morphology and biological functions. Recent studies have confirmed that sclerostin expression in oral tissue is not only in osteocytes of alveolar bone, but also in odontoblasts and dental pulp stem cells (DPSCs) in dental pulp, cementocytes in cementum, and periodontal ligament cells (PDLCs) (Fig. 2; Table 1). Additionally, sclerostin is also distributed in gingival tissue and gingival crevicular fluid. The wide range of sclerostin expression in oral tissues and cells has shown regulatory potential of sclerostin in dental homeostasis.

**Table 1:** The expression of sclerostin in oral tissues and cells

Cell/tissue	Species	Detection methods	Refs.
Gingival crevicular fluid	Human	ELISA/qPCR	[32–36]
Gingiva	Human	ELISA/qPCR	[37, 38]
Periodontal ligament cell	Human	Western Blot/qPCR Immunohistochemistry Immunofluorescent staining	[39–41]
	Mouse	Confocal microscopy	[42]
Osteocytes in alveolar bone	Human	Immunohistochemistry	[39, 43]
	Mouse	LacZ staining/qPCR Immunohistochemistry Immunofluorescent staining	[39, 42, 44–47]
	Rat	Immunohistochemistry	[48–51]
Odontoblast	Human	Immunohistochemistry	[52, 53]
	Mouse	Immunohistochemistry	[45, 54]
Odontoblast-like cell	Human	Western blot/qPCR	[52, 55]
	Mouse	Immunohistochemistry	[56]
Dental pulp stem cell	Human	Western blot/qPCR	[53, 55, 57]
	Mouse	qPCR	[56]
Cementocyte	Human	Immunohistochemistry qPCR	[39, 58]
	Mouse	Immunohistochemistry LacZ staining	[39, 44, 59, 60]

*ELISA* enzyme-linked immunosorbent assay; *qPCR* quantitative real-time polymerase chain reaction

## Role of sclerostin in oral hard tissues

### Sclerostin and alveolar bone remodeling under mechanical stress

Mechanical stress stimulation is a crucial factor that regulates bone growth and bone remodeling [61, 62]. Osteocytes can perceive external stimuli via the primary cilia, cell membrane, and dendrites [29, 63]. Sclerostin secreted by osteocytes serves as a mediator of mechanotransduction, characterized by the significant enhancement of the serum levels of sclerostin in patients with disused osteoporosis caused by unloading [64]. Several in vivo and in vitro studies have demonstrated that sclerostin can act as a mediator between osteocytes and osteoblasts to inhibit osteoblasts mineralization. Consequently, mechanical loading could reduce sclerostin expression to promote bone formation in wild-type mice, but *SOST* knock-out mice were insensitive to unloading and did not have bone loss [14, 15]. Therefore, it has been recognized that under mechanical stress, sclerostin exerts negative regulatory effects on bone formation.

Alveolar bone, a highly plastic tissue surrounding the root, is the crucial supporting structure of tooth. *SOST* knock-out mice had higher density and volume of mandibular alveolar bone than wild-type mice, and systemic Scl-Ab administration for rats with alveolar bone defects could improve bone regeneration, suggesting the essential role of sclerostin in the metabolism of alveolar bone [65, 66]. Alveolar bone is the most actively remodeled bone tissue throughout the body, and alveolar bone loss is related to age, menopause, periodontal disease, and change of occlusal stress [67, 68]. Disused loss of alveolar bone induced by the reduction or loss of occlusal stress resembles disused osteoporosis, involving local and systemic multiple factors. In a recent study, the maxillary molar of rats was pulled out to simulate occlusal stress reduction. It was found that *SOST* mRNA was remarkably upregulated in the osteocytes of the opposing mandible alveolar bone, and Scl-Ab treatment could significantly increase the bone mass of opposing alveolar ridge [49], thereby revealing that sclerostin is involved in alveolar bone remodeling after occlusal stress loss.

Orthodontic tooth movement (OTM) is the process in which exogenous mechanical force acting on teeth is transmitted to the periodontal tissues leading to alveolar bone remodeling. During OTM, the anabolism of alveolar bone is highly active on the tension side, whereas catabolism is dominant on the compression side [69]. Related studies often use Ni–Ti closed-coil springs holding the upper bilateral incisors and unilateral first molar of rodents to simulate OTM in vivo. The results demonstrated that orthodontic force could modulate the expression level of sclerostin in alveolar bone (Table 2). Sclerostin was often decreased on the tension side, whereas, on the compression side, sclerostin showed a significant increase at the early stage and then gradually dropped back to the initial level [42, 46, 48]. Oxygen concentration is one of the factors regulating the sclerostin expression level [17], hence, the difference of dynamic trends between the tension and compression sides might be related to the ischemic hypoxic condition of the compression side. Moreover, the dynamic trends corresponded with the negative role of sclerostin in bone mass and the remodeling process at the tension/compression site during OTM. In another study setting up a relapse group, OTM was removed after one week and the molar was allowed to relapse for another week. The results showed that when compared to the control group (receiving OTM for 2 weeks), sclerostin expression was increased on both sides (tension and compression) followed by decreased alveolar bone mass [47], validating sclerostin’s role in alveolar bone remodeling. Additionally, in *SOST* knock-out mice, the expression of RANKL and the number of osteoclasts in alveolar bone were lower than those in wild-type mice during OTM (force: 5.1 g) [48]. These results infer that sclerostin is vital in alveolar bone remodeling during OTM via both osteogenic and osteoclastic responses.

**Table 2:** Sclerostin expression alteration in alveolar bone during orthodontic tooth movement

Animal	Force (g)	Time (days)	Sclerostin level		Detection method	Refs.
			Tension side	Compression side		
Mouse	10	4	↓	↑	Confocal fluorescence imaging	[42]
Rat	20	1	↓	↑	Immunohistochemistry	[48]
		7	↓	→		
		28	↓	↓		
Mouse	10	1	↓	↓	Immunofluorescence	[46]
		5	→	↑		
		10	→	→		
Mouse	10	14	↑^a^	↑^a^	Immunohistochemistry	[47]

(Results are generally compared with day 0, but ^a^ compared with relapse group)

PDLCs in periodontal ligament are pluripotent stem cells with osteogenic potential, which are the main source of osteoblasts responsible for forming alveolar bone [70]. Hence, many related researches also use PDLCs as the in vitro model to investigate alveolar bone remodeling under mechanical stress. Several studies have confirmed that PDLCs were highly sensitive to mechanical stimulation, and the tension force could elevate the expression of osteogenic marker genes in PDLCs to promote the osteogenic differentiation [71–74]. The mechanosensory ability of PDLCs was regulated by osteocytes-derived sclerostin, and *SOST* knock-out or inhibition in mice would eliminate the response of PDLCs to mechanical force [44, 75]. Sclerostin was also expressed in PDLCs cultured from human periodontal ligament and the intermittent compressive force (1.5 g/cm^2^, 14 rounds/min) could improve sclerostin expression in PDLCs [40]. Another study found that when mild compressive force (0.24 g/cm^2^) was applied to PDLCs, the expressions of sclerostin and RANKL decreased, whereas they increased when severe compressive force (2.4 g/cm^2^) was applied [46]. Accordingly, sclerostin may regulate the osteoclastic function of PDLCs to act on alveolar bone resorption under compressive stress, but more direct experimental evidence is still needed. In addition to compression, mechanical stress also involves other factors, such as tension, fluid shear stress and so on. Relevant researches will need to be further complemented and improved in the future.

Above all, mechanical stress can regulate sclerostin expression in osteocytes of alveolar bone and PDLCs, and sclerostin may participate in alveolar bone remodeling by directly or indirectly regulating the balance of bone formation and bone resorption.

### Sclerostin and dentinogenesis

Dentin is the core tissue of tooth, which supports the enamel on the outside and protects the pulp on the inside. It resembles bone in the mesenchymal origin, composition, and especially biomineralization mechanisms that mainly involve collagen formation and matrix mineralization. Odontoblasts act as the most crucial part of dentinogenesis by synthesizing dentin matrix and regulating its mineralization. After finishing the terminal differentiation, odontoblasts can secrete type I collagen matrix, non-collagen, and proteoglycans, and then promote the transport of calcium and inorganic phosphate to the mineralization front [76, 77]. Odontoblasts are similar to osteocytes not only in their mesenchymal origin and mechanosensory ability, but also in sclerostin secretion. An experiment on fetal mice found that sclerostin was expressed in the secretory odontoblasts of tooth germs, thus demonstrating the potential correlation between sclerostin and dentin growth [45]. Another study on postnatal mice also confirmed the positive sclerostin expression in odontoblasts, and demonstrated that the physiological downregulation of sclerostin along with the inhibition of osteoclast activity might initiate the growth transitions from primary dentin to secondary dentin and from crown to root [54]. Besides, sclerostin expression was interacted with the expression of dentin sialophosphoprotein, a critical protein for dentin mineralization [52, 53, 78].

**Fig. 3:**
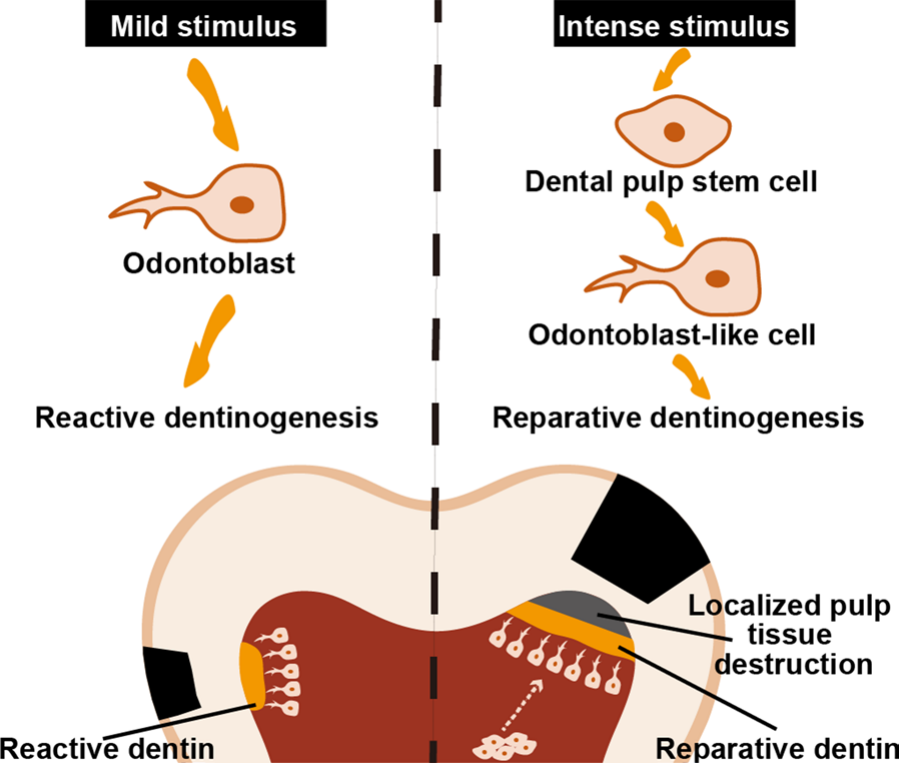
Schematic diagram of tertiary dentin formation. Reactive dentin is secreted by surviving odontoblasts under mild stimulus, whereas reparative dentin is secreted by newly formed odontoblast-like cells that originate from dental pulp stem cells in response to intense stimulus

The tertiary dentin, subclassified as either reparative or reactive, is newly formed after tooth eruption caused by external stimuli such as abrasion, trauma, and caries [79]. Following mild stimuli, surviving odontoblasts are activated and secrete reactive dentin; upon intense stimulation, local odontoblasts would die and DPSCs in dental pulp would differentiate into odontoblast-like cells to form the reparative dentin [80] (Fig. 3). Non-carious sclerotic dentin is a kind of reactive dentin mainly located beneath non-carious cervical lesions (NCCLs), for instance, wedge-shaped defects. Closed tubules and hyper-mineralized matrix contribute to sclerotic dentin as a protective barrier that prevents external stimulation into the pulp cavity [81, 82]. The precise forming mechanisms of sclerotic dentin still remains undefined. After all, mechanical stress exerted by transverse toothbrushing and occlusal over-loading, the fundamental etiology of NCCLs, should be an underlying inducing cause of sclerotic dentin formation [83–85]. Previous study of the author collected human teeth with NCCLs and cultured human odontoblasts in vitro, and found that sclerostin expression decreased in odontoblasts beneath NCCLs, and that sclerostin overexpression in odontoblasts inhibited the odontogenic differentiation under tensile stress, suggesting that sclerostin might participate in non-carious sclerotic dentin formation under mechanical stress by inhibiting the differentiation of odontoblasts [52]. DPSCs are the sole source of regenerative odontoblasts in vivo. Recent research found that sclerostin could impair the odontoblastic potential of DPSCs, thus indicating the negative role of sclerostin in reparative dentinogenesis [53]. Another study on reparative dentin established a pulp injury model in mice by exposing the pulp of maxillary first molars with a metal file and covered with mineral trioxide aggregate (MTA) materials. The results showed that sclerostin was expressed in newly formed odontoblast-like cells, and the forming rate of reparative dentin was faster in *SOST* knock-out mice than that in wild-type mice [56].

Tertiary dentin holds great significance for tooth development and pulp repair. For severely damaged pulp, applying tissue engineering technology to achieve pulp/dentin regeneration has become a research hotspot and clinical trend, which is also based on the odontogenic ability of transplanted DPSCs. Up till the present studies, sclerostin has shown similar inhibitory effects on dentinogenesis as osteogenesis. In the view of the breakthrough that anti-sclerostin becomes a new target for osteoporosis treatment, future studies on the regulatory function of sclerostin on dentin biomineralization may promote the development of dentin repair and pulp regeneration. Eventually, novel strategies for preventing and treating dentin diseases such as dentin hypersensitiveness and dentin defects will evolve.

### Sclerostin and cementogenesis

Cementum, another bone-like tissue of tooth, is the bridge connecting tooth and periodontal tissues to maintain the tooth stability and periodontal health. Cementum can be divided into cellular cementum and acellular cementum, with cellular cementum having a regenerative capacity. The cellular cementum layer covering the root surface plays a protective role in root maintenance and cementum regeneration is crucial to the repairment of external root resorption under physiological or pathological condition.

Sclerostin is expressed in human and mouse cementocytes [39, 58, 59]. Patients with sclerosteosis or Van Buchem disease have a phenotype of increased cementum [86], and *SOST* gene deficiency in mice also induces buccal and lingual cementum thickening [65], which reflects the inhibitory effect of sclerostin on cementogenesis. Another study in mice found that sclerostin was not detected at the initial stage of cementogenesis, but was expressed in the apical cellular cementum at four weeks old, and the expression is elevated at 8 weeks old, suggesting that sclerostin might be involved in the regulation of cementum homeostasis and cementum regeneration [59]. Furtherly, a recent study experimentally created osseous defects around teeth in rats and found that the systemic administration of Scl-Ab could increase cementogenesis to improve cemental repair [66].

Orthodontic treatment aims to correct the teeth arrangement, but induces a severe side effect of root resorption on the compressive side during OTM [87]. Cementoblasts are essential effector cells for root protection that not only resist the attachment of cementoclasts, but also secret new cementum-like tissue to repair the absorbed root. In vitro studies have shown that sclerostin could inhibit the proliferation and differentiation of cementoblasts, and promote root resorption under compressive force by modulating the RANKL/OPG axis [88–91].

Cementogenesis is crucial for root development and repairment. Further identification of sclerostin’s negative effects on cementum formation and regeneration will benefit the treatment of cementum-related diseases, especially OTM-induced root resorption.

## Role of sclerostin in oral inflammation

### Sclerostin and pulp inflammation

Dental pulp inflammation is often a sequel to caries or trauma that facilitates the penetration of pathogens and their bacterial products. Odontoblasts occupy a particular location in the dentin-pulp interface, and their cytoplasmic processes extend along the dentinal tubules to sense the external stimulation. Consequently, odontoblasts serve as the first cellular line of host defense exposed to pathogens and initiate the pupal inflammatory immune responses by secreting various cytokines and chemokines [92–94]. However, odontoblasts possess limited anti-inflammatory ability under intense stimulation, and then DPSCs would migrate to the injury site and differentiate into odontoblast-like cells to generate a reparative dentin barrier [95, 96]. The objective of vital pulp therapy is to replace odontoblasts with DPSCs, which has now become a preferable treatment for reversible and localized pulpitis.

Sclerostin has been proposed as an inflammatory regulator based on the experimental findings that sclerostin inhibition can prevent bone loss in patients with rheumatoid arthritis and colitis [97–99]. In DPSCs, inflammatory cytokine interleukin-1β could downregulate sclerostin expression at the transcription level [57]. A previous in vitro study of the author found that in lipopolysaccharide-induced inflammatory condition, sclerostin promoted the production of several critical pro-inflammatory cytokines in odontoblasts and inhibited the odontoblastic differentiation of inflamed DPCs, revealing that sclerostin might play a pro-inflammatory role in dental pulp inflammation and impair dentin regeneration [55].

Inflammation and aging often interact in dental pulp: inflammation can induce oxidative stress and DNA damage to cause the premature senescence of dental pulp cells (DPCs); aging is also accompanied by chronic inflammation because of the upregulated production of inflammatory molecules [100–103]. Recent studies have reported significantly elevated sclerostin in the serum and pulp tissue of the elderly, and sclerostin could induce DPCs senescence in vitro [53, 104, 105]. Hence, sclerostin might also participate in the regulation of dental pulp inflammation by promoting the aging process of DPCs.

If pulp infection evolves into pulpitis or apical periodontitis, the patients will suffer severe pain and even tooth loss. The precise procedure and regulation of dental pulp inflammation are yet to be well elucidated. Existing experimental evidence suggests that sclerostin inhibition seems to be promising for anti-inflammation and pro-regeneration during dental pulp inflammation. More convincing evidence and extensive exploration of applications are still needed.

### Sclerostin and periodontal disease

Periodontal disease, one of the essential reasons causing tooth loss, refers to the chronic inflammation of periodontal supporting tissues caused by local factors, and includes gingivitis and periodontitis. Once gingivitis deteriorates to periodontitis, it will manifest as attachment loss, alveolar bone resorption and pathological periodontal pocket formation. Sclerostin has been shown to play a pro-inflammatory role in arthritis and colitis [97, 98, 106]. With the discovery of positive sclerostin expression in PDLCs [59], its role in periodontitis has recently become a popular subject to explore. Clinical studies have shown that sclerostin level is higher in the gingival tissue, gingival crevicular fluid and serum of patients with chronic periodontitis than in healthy people, and is accompanied by an increased RANKL production [32, 33, 36–38]. Sclerostin serves as positive feedback for RANKL expression in inflammation progression, implying that sclerostin may have a promoting effect on periodontitis, furthermore, osteoclast might be conductive to this promotion process.

Alveolar bone loss is the critical pathological manifestation of periodontitis. Periodontitis often leads to tooth loosening and even tooth loss due to the destruction of periodontal supporting tissues. The influence of periodontitis on alveolar bone remodeling is not only reflected in occlusal stress change (described in preceding section), but more importantly, in local inflammation-caused bone resorption [107]. Establishing a periodontitis model in rats by ligating the first molar with silk thread, researchers found that sclerostin expression in the alveolar bone was higher in rats with periodontitis, and furtherly, increased sclerostin along with decreased osteogenesis in the early stage of periodontitis, then decreased sclerostin followed by more osteoid formation in the later stage [50, 51, 108]. Furthermore, rats with periodontitis who received treatment with Scl-Ab showed a rebound in the alveolar crest level, alveolar bone mass and the serum expression level of osteocalcin and osteopontin [109]. Thus, it can be seen that sclerostin in alveolar osteocytes plays a crucial role in alveolar bone formation during periodontitis.

Periostin is an essential matrix protein for periodontal ligament formation. Periostin is highly expressed in the periodontal ligament, but the periostin level of gingival crevicular fluid in periodontitis patients was lower than in healthy people [110, 111]. Periostin deficiency destroys the integrity of periodontal ligament, further leading to alveolar bone loss, periodontal tissue inflammation, periodontal pocket formation and other symptoms similar to periodontitis [112]. However, the offspring of *SOST* knock-out mice and periostin null mice did not have alveolar bone loss, and an early treatment of Scl-Ab to periostin null mice could prevent the changes of osteocytes morphology and the appearance of periodontitis symptoms, and a late treatment could increase the density and height of alveolar bone and reduce the formation of bone resorption lacunae [113]. These results validate that sclerostin acts as a regulatory mediator of periostin and periodontal home homeostasis.

**Fig. 4:**
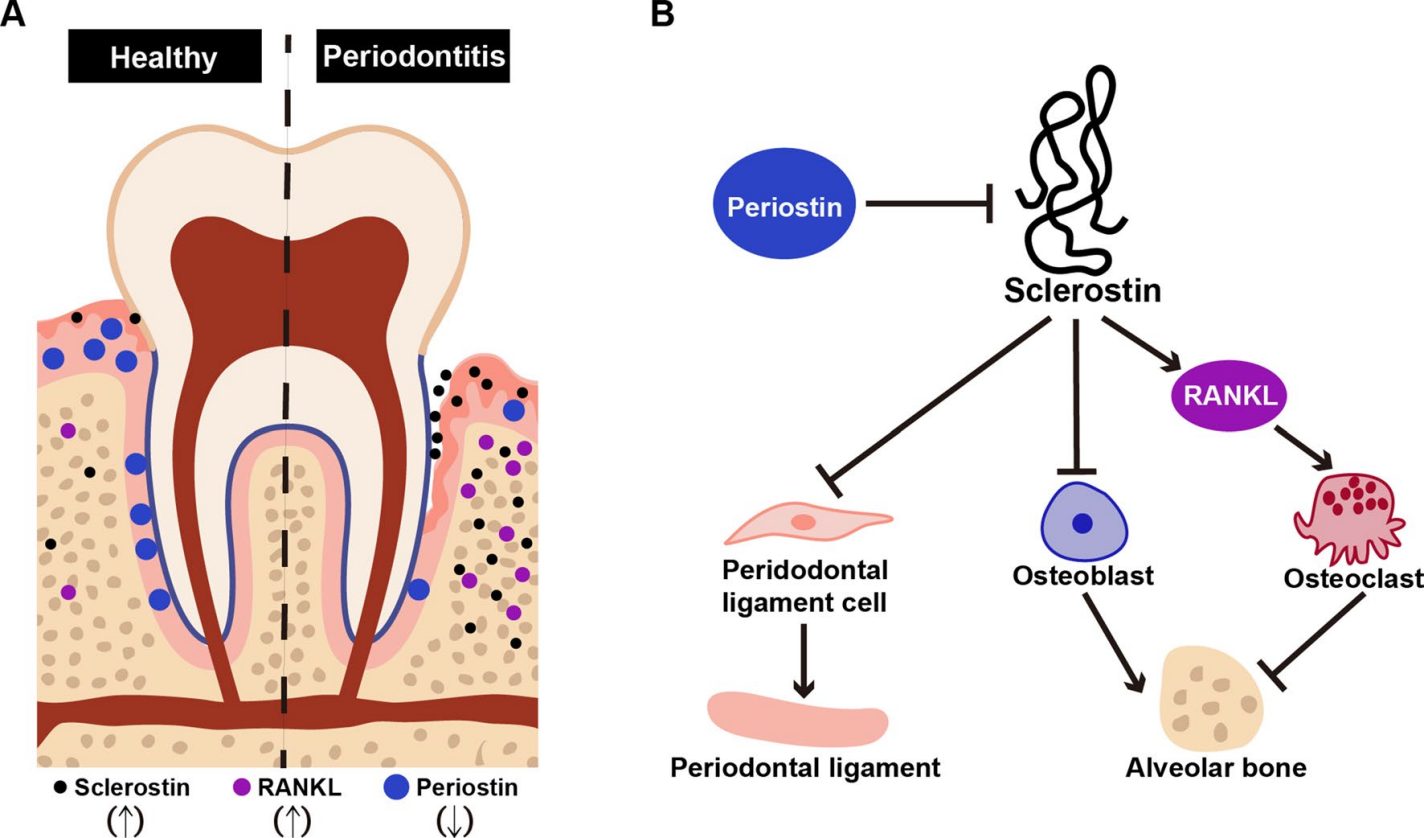
Current understanding about the role of sclerostin in periodontitis. **A** In periodontitis condition, the expression of sclerostin and RANKL is increased while the expression of periostin is decreased. **B** Sclerostin participates in periodontitis development by regulating periodontal ligament morphology and alveolar bone metabolism. *RANKL* receptor activator of nuclear factor-κB ligand

In summary, current studies demonstrate that sclerostin may contribute to the development of periodontitis by regulating the anabolism and catabolism of alveolar bone, and sclerostin inhibition during periodontitis could improve alveolar bone mass and repair the morphology of periodontal ligament (Fig. 4). The detection of sclerostin level and the application of Scl-Ab offer great potential for the diagnosing and treating periodontitis.

## Role of sclerostin in dental implantation

### Sclerostin and osseointegration

Dental implantation is a reliable treatment for repairing missing tooth in oral clinical treatment. Osseointegration is defined as the formation of a direct interface between living bone and a load-carrying implant without intervening soft tissue, which is the basic theory and pursue goal of dental implantation. Adequate bone mass at the edentulous ridge is fundamental for osseointegration after dental implant placement. The challenge of stable osseointegration lies in how to accelerate alveolar bone regeneration to shorten implant healing time and maintain long-term stability.

Because sclerostin serves as a principal regulator of bone metabolism, its potential role on bone-implant osseointegration has generated extensive interest in the oral field. The application of Scl-Ab on rats with femoral implants could enhance implants retention by promoting the osteogenesis of cortical bone and bone trabecula [114, 115]. To better mimic dental implantation in the oral environment, customized small implants were implanted in the rat alveolar bone one month after the extraction of maxillary first molar, and the results showed that Scl-Ab treatment could significantly increase maxilla bone mass and promote implant osseointegration [116].

Osteoporosis is a potential risk factor for the failure of dental implantation because of the difficulties on bone healing and osseointegration forming. Recent research found that sclerostin deficiency increased bone regeneration within rodent calvarial defect [117], and sclerostin impaired microRNA-based tissue engineering therapy for canine mandibular defect [118], inferring positive outcomes of sclerostin inhibition on alveolar bone augmentation. Moreover, several studies reported that *SOST* knock-out or Scl-Ab treatment could significantly promote the osseointegration of femoral implants and tibial implants in ovariectomized osteoporotic rodents [119–121]. These findings support the view that sclerostin inhibition might be a promising therapy to promote implant osseointegration for patients with insufficient bone mass or bone defects.

### Sclerostin and peri-implant diseases

Implant dentures provide similar esthetics and chewing functionality to natural teeth. However, the broad application of implants makes peri-implant diseases become a serious clinical challenge. The inflammatory change of soft tissue around implants is called peri-implant mucositis, and the alveolar bone defect caused by peri-implant mucositis is called peri-implantitis. The prevention and treatment of peri-implant diseases are increasingly important for oral clinicians.

In a broad sense, both peri-implant mucositis and peri-implantitis belong to periodontal disease group. Two clinical studies collecting gingival crevicular fluid of patients with implants showed that the sclerostin level is significantly higher around inflamed implants than around healthy implants, suggesting that sclerostin might serve as a valuable biomarker of peri-implantitis [34, 35]. Furthermore, Scl-Ab treatment for rats with femoral implants could prevent aseptic implant loosening and osteolysis around implants [114, 115]. Accordingly, as a potential regulator of periodontitis, sclerostin may also exert a pro-inflammatory effect on peri-implant diseases.

## Conclusions and future perspectives

Since the discovery of *SOST* gene in the late 1990s, the role and mechanisms of sclerostin on bone metabolism have been extensively investigated. The monoclonal antibody romosozumab has been approved as an option for patients with osteoporosis, and is promising for treating other bone diseases such as fracture, sclerosteosis and myeloma bone disease. These clinical applications inspired the therapeutic exploration of sclerostin in clinical fields other than bone. In recent years, with the positive sclerostin expression detected in oral tissues and cells, its function in dentistry has gradually been explored and recognized.

In conclusion, sclerostin regulates hard tissue formation and inflammatory diseases in the oral cavity. As a bone-modifying regulator, sclerostin is an inhibitor of alveolar bone mass by regulating the balance of anabolism and catabolism, proceeding to affect the progress of periodontitis and bone-implant osseointegration. For bone-like tissue, sclerostin plays a similar negative role in dentin formation and cementum regeneration, regulating pulp/dentin regeneration after pulp extirpation and root resorption during orthodontic treatment. As an inflammatory regulator, sclerostin may exert pro-inflammatory effects in oral inflammatory diseases, and its expression level has the potential to become a diagnostic biomarker of gingivitis, periodontitis, pulpitis and peri-implantitis. However, some of existing research progress only provides preliminary proof via in vitro experiments or human source sample detection. More convincing in vivo evidence and related mechanistic investigation are required.

Future research works on sclerostin in the oral and dental fields may focus on the following aspects: (1) enamel is another bone-like tissue of tooth and is crucial for tooth defense, therefore, the research gap of the effects of sclerostin on enamel formation should be filled; (2) tissue engineering and regenerative medicine based on stem cell transplantation have become frontier research topics in recent years; the effects of sclerostin on a variety of stem cell types in oral tissues (such as DPSCs, PDLCs, dental follicle stem cells, stem cells from apical papilla, etc.) should be further improved and supplied for promoting the development of regenerative dentistry; (3) most rodent experiments about Scl-Ab and alveolar bone used systemic administration by subcutaneous injection, but the effects of Scl-Ab on whole-body bone were barely mentioned; among these previous studies, one study compared systemic and local administration of Scl-Ab and found local delivery had no healing role on alveolar bone defect [66]; hence, before the application of Scl-Ab in oral and dental clinical treatment, the mode of administration and its systemic influence should be further studied; (4) clinical trials of romosozumab showed increased potential cardiovascular risk [122, 123], and sclerostin level is related to chronic kidney disease [124, 125]; thus, in future studies about sclerostin and oral diseases, more attention should be paid to the relationship with general systemic disease.

Teeth play vital roles in life. Although the underlying mechanisms of sclerostin in the oral cavity have not been fully elucidated, its functions on the metabolism of alveolar bone, dentin and cementum along with its oral inflammation regulation have become increasingly apparent. Sclerostin inhibition in particular effector cells through Scl-Ab administration has the potential to become a therapeutic target for dental indications such as periodontitis, pulpitis, orthodontic complications, and dental implantation, among others. Further research for complete understanding of sclerostin’s biology and pathophysiology has broad prospection and essential clinical significance.

## Data Availability

Not applicable.

## Abbreviations

BMP: Bone morphogenetic protein
DAN: Differential screening-selected gene aberrative in neuroblastoma
DPSC: Dental pulp stem cell
OPG: Osteoprotegerin
OTM: Orthodontic tooth movement
PDLC: Periodontal ligament cell
RANKL: Receptor activator of nuclear factor-kappaB ligand
Scl-Ab: Sclerostin-neutralizing antibody
